# The potential of precision diabetology for type 2 diabetes treatment—evidence from a meta-regression for all-cause mortality from large cardiovascular outcome trials

**DOI:** 10.1007/s00592-024-02425-8

**Published:** 2024-12-12

**Authors:** Oliver Kuss, Michael Roden, Sabrina Schlesinger, Annika Hoyer

**Affiliations:** 1 Institute for Biometrics and Epidemiology, German Diabetes Center, Leibniz Center for Diabetes Research, Heinrich Heine University Düsseldorf, Auf’m Hennekamp 65, 40225 Düsseldorf, Germany; 2 Centre for Health and Society, Faculty of Medicine, Heinrich Heine University Düsseldorf, Düsseldorf, Germany; 3 German Center for Diabetes Research, Partner Düsseldorf, München-Neuherberg, Germany; 4 Institute for Clinical Diabetology, German Diabetes Center, Leibniz Center for Diabetes Research, Heinrich Heine University Düsseldorf, Düsseldorf, Germany; 5 Department of Endocrinology and Diabetology, Medical Faculty and University Hospital Düsseldorf, Heinrich Heine University Düsseldorf, Düsseldorf, Germany; 6 Biostatistics and Medical Biometry, Medical School OWL, Bielefeld University, Bielefeld, Germany

**Keywords:** Dipeptidyl peptidase-4 inhibitors, Glucagon-like peptide 1, HbA_1c_, Meta-regression, Precision medicine, Sodium–glucose transporter 2 inhibitors, Type 2 diabetes mellitus

## Abstract

**Aims:**

Two prerequisites must be met for the precision treatment approach to be beneficial for treated individuals. First, there must be treatment heterogeneity; second, in case of treatment heterogeneity, clinical predictors to identify people who would benefit from one treatment more than from others must be available. There is an established meta-regression approach to assess these two prerequisites that relies on measuring the variability of a clinical outcome after treatment in placebo-controlled randomised trials. We recently applied this approach to the treatment of type 2 diabetes for the clinical outcomes of glycaemic control and body weight and repeat it for the clinical outcome of all-cause mortality.

**Methods:**

We performed a meta-regression analysis using digitalized individual participant information on time to death from 10 large cardiovascular outcome trials (7563 deaths from 99,746 participants) on DPP-4 inhibitors, GLP-1 receptor agonists, and SGLT-2 inhibitors with respect to the variability of all-cause mortality and its potential predictors after treatment.

**Results:**

The adjusted difference in log(SD) values of time to death between the verum and placebo arms was −0.036 (95%-CI: −0.059; −0.013), showing larger variability of time to death in the placebo arms. No clinical predictors were found to explain treatment heterogeneity.

**Conclusions:**

This analysis suggests that the potential of the precision treatment approach in type 2 diabetes is low, at least with regard to improvement of all-cause mortality in population with high cardiovascular risk. This extends our previous findings for the clinical outcomes of glycaemic control and body weight.

**Supplementary Information:**

The online version contains supplementary material available at 10.1007/s00592-024-02425-8.

## Background

The idea of precision treatment, i.e. providing the right treatment for the right individual at the right time, is very current in diabetes research and is driven by the hope of optimizing care and ultimately improving quality of life for all people with diabetes. This is, for example, reflected in the fact that the two leading diabetes societies, the ADA and the EASD, founded a “Precision Medicine in Diabetes Initiative” in 2018, issued a common consensus report [1], two updates [2, 3], and commissioned a number of systematic reviews on the topic, e.g. on treatment effect heterogeneity for GLP1-receptor agonists and SGLT2-inhibitors [4]. Only recently, the National Institute of Diabetes and Digestive and Kidney Diseases (NIDDK) founded a global initiative to deliver precision health in diabetes, which assessed available approaches to disease heterogeneity and identified current research gaps [5].

There is an established meta-regression method to empirically evaluate the precision treatment approach with respect to its clinical benefit for an individual. In general, two prerequisites must be met for precision treatment to be clinically useful. First, there must be treatment heterogeneity, that is, the same individual should react differently to different treatments. Second, in case of treatment heterogeneity, there should be clinical predictors to identify treatments that work better than others in this treated individual. The meta-regression method to assess these two prerequisites relies on measuring the variability of a clinical outcome after treatment in placebo-controlled randomized trials. We recently showed the applicability of this approach for the clinical outcomes of glycemic control and body weight [6, 7].

The aim of this work is to investigate the potential benefit of the precision treatment approach for the clinical outcome of all-cause mortality. Thus, we use meta-regression analysis with digitalized individual participant information on time to death from cardiovascular outcome trials on DPP-4 inhibitors, GLP-1 receptor agonists, and SGLT-2 inhibitors.

## Methods

We downloaded full texts and online supplements of cardiovascular outcome trials (CVOT) as given in the regular annual CVOT summit reports from Schnell et al. [8–15], which (1) reported on a population with diabetes, (2) compared a DPP-4 inhibitor, a GLP-1 receptor agonist, or an SGLT-2 inhibitor to placebo, and (3) allowed to digitalize data on all-cause mortality from published Kaplan–Meier curves. To identify CVOTs outside the regular CVOT summit reports, we additionally checked two frequently cited reviews of CVOTs [16, 17]. With respect to the definitions of diabetes, we had to rely on those from the original trials. In general, these trials (following the requirements of the U.S. Food and Drug Administration) used populations with type 2 diabetes and established cardiovascular disease or risk factors.

We used WebPlotDigitizer, Version 4.2 [18], and the R code of Guyot et al. [19] to extract individual participants´ information on time to death or censoring from the Kaplan-Meier curves in the original trial populations. Both methods have been shown to be reliable and valid by others [20] and also in our previous work [21, 22].

To achieve estimates for the variability of all-cause mortality, we fitted Weibull distributions (Additional file, Table S1) separately in all verum (i.e. active treatment) and placebo arms to the extracted data. As the actual variability outcome for time to death we used the natural logarithm of the standard deviation (log[SD]) of the times to death, following the recommendations of Nakagawa et al. [23] for continuous responses.

To assess treatment heterogeneity, we fitted a weighted meta-regression model with the single trial arms being the observational units. This model had the log(SD) of time to death as the outcome, the treatment (verum vs. placebo) as the covariate of central interest, the natural logarithm of the mean (log[Mean]) of time to death as a second covariate and a random intercept for each trial to account for the correlation of arms within trials. An estimated regression coefficient of 0 for the treatment covariate in this meta-regression model indicates that the variability (i.e. log(SD)) of time to death is equal in verum and placebo groups, suggesting that there is no potential for the precision treatment approach. To account for different sample sizes in arms and trials, the meta-regression model weighted each observation by the inverse of the estimated variance of the log(SD).

For assessing the existence of clinical predictors to explain treatment heterogeneity, we used a separate meta-regression model for each clinical predictor. The meta-regression model as described in the previous paragraph was extended by an additional interaction term of the respective clinical predictor with treatment. Evaluated continuous clinical predictors were mean age, proportion of male participants, mean BMI, mean HbA1c, mean disease duration, mean eGFR, mean systolic blood pressure, mean total cholesterol, mean triglycerides (all of them as measured at baseline), and median follow-up time. In addition, we assessed the year in which the trial was performed as a potential continuous clinical predictor, and the drug class as a categorical clinical predictor.

We used SAS (SAS Institute Inc., Cary, NC, USA), Version 9.4, for data management and analysis.

## Results

We achieved the full individual time-to-event information with respect to all-cause mortality from 3 trials on DPP-4 inhibitors (CARMELINA, EXAMINE, TECOS), 3 on GLP-1 receptor agonists (EXSCEL, LEADER, REWIND) and 4 on SGLT-2 inhibitors (CANVAS, CREDENCE, DECLARE-TIMI 58, EMPA-REG Outcome), see Table 1. DAPA-CKD, DAPA-HF, and EMPEROR-REDUCED were excluded due to their trial populations containing also individuals without diabetes. VERTIS-CV, SAVOR-TIMI, HARMONY-O, AMPLITUDE-O, SOLOIST, and SCORED did not report Kaplan-Meier curves in their publications. The FIGARO-DKD was excluded because it assessed Finerenone, a mineralocorticoid receptor antagonist.

**Table 1:** Description of included trials, separately for placebo and verum arms

Study	Drug class	Number of deaths	Number of obser- vations	Median follow-up time (in months)	Year	Mean age at baseline (in years)	Proportion of male participants at baseline (in %)	Mean BMI at baseline	Mean HbA1c at baseline (in %)	Mean HbA1c at baseline (in mmol/ mol)	Mean disease duration at baseline (in years)	Mean eGFR at baseline (in mL/min/1.73 m^2^)	Mean systolic BP at baseline (in mmHG)	Mean total cholesterol at baseline (in mg/dL)	Mean triglycerides at baseline (in mg/dL)
*Placebo*															
CANVAS	SGLT2	289	4347	28.1	2017	63.4	63.3	32.0	8.2	66.1	13.7	76.2	136.9	170.2	177.0
CARMELINA	DPP-4	373	3485	26.5	2018	65.6	64.3	31.3	8.0	63.9	14.5	54.5	140.6	171.0	187.0
CREDENCE	SGLT2	200	2199	31.3	2019	63.2	66.7	31.3	8.3	67.2	16.0	56.0	140.2	179.8	197.0
DECLARE-TIMI 58	SGLT2	489	8578	47.3	2019	64.0	62.1	32.0	8.3	67.2	10.0	85.1	134.8	–	–
EMPA-REG Outcome	SGLT2	194	2333	37.5	2015	63.2	72.0	30.7	8.1	64.8	–	73.8	135.8	161.9	170.7
EXAMINE	DPP-4	166	2679	19.4	2013	61.0	67.7	28.7	8.0	63.9	7.1	71.1	–	154.8	166.4
EXSCEL	GLP-1	564	7396	39.8	2017	62.0	62.0	31.7	8.0	63.9	12.0	76.0	–	–	–
LEADER	GLP-1	445	4672	46.5	2016	64.4	64.0	32.5	8.7	71.6	12.9	80.6	135.9	–	–
REWIND	GLP-1	585	4952	65.4	2019	66.2	53.9	32.3	7.4	57.4	10.6	76.6	137.3	174.8	141.6
TECOS	DPP-4	518	7339	36.1	2015	65.5	70.5	30.2	7.2	55.2	11.6	74.9	135.0	165.4	164.8
*Verum*															
CANVAS	SGLT2	412	5795	33.9	2017	63.2	64.9	31.9	8.2	66.1	13.5	76.7	136.4	170.2	177.0
CARMELINA	DPP-4	366	3494	26.8	2018	66.1	61.5	31.4	7.9	62.8	15.0	54.7	140.4	173.0	190.0
CREDENCE	SGLT2	166	2202	31.6	2019	62.9	65.4	31.4	8.3	67.2	15.5	56.3	139.8	180.9	198.8
DECLARE-TIMI 58	SGLT2	465	8582	47.3	2019	63.9	63.1	32.1	8.3	67.2	11.0	85.4	135.1	–	–
EMPA-REG Outcome	SGLT2	267	4687	37.9	2015	63.1	71.2	30.6	8.1	64.7	–	74.2	135.3	163.5	170.5
EXAMINE	DPP-4	150	2701	19.5	2013	61.0	68.0	28.7	8.0	63.9	7.3	71.2	–	153.9	162.7
EXSCEL	GLP-1	487	7356	40.1	2017	62.0	62.0	31.8	8.0	63.9	12.0	76.6	–	–	–
LEADER	GLP-1	379	4668	46.7	2016	64.2	64.5	32.5	8.7	71.6	12.8	80.2	135.9	–	–
REWIND	GLP-1	524	4949	65.3	2019	66.2	53.4	32.3	7.3	56.3	10.5	77.2	137.1	174.8	141.6
TECOS	DPP-4	524	7332	36.4	2015	65.4	70.9	30.2	7.2	55.2	11.6	74.9	135.0	166.1	166.0

In the ten included CVOTs, a total of 7563 deaths from 99,746 observations were observed, 3823 deaths from 47,980 observations in the placebo arms and 3740 deaths from 51,766 observations in the verum arms, indicating reduced all-cause mortality in the verum groups. According to previous work [21], using the Weibull distribution for the survival curves resulted in near perfect fits to the Kaplan–Meier curves from the digitalized data (see Additional file, Fig. S1).

**Fig. 1:**
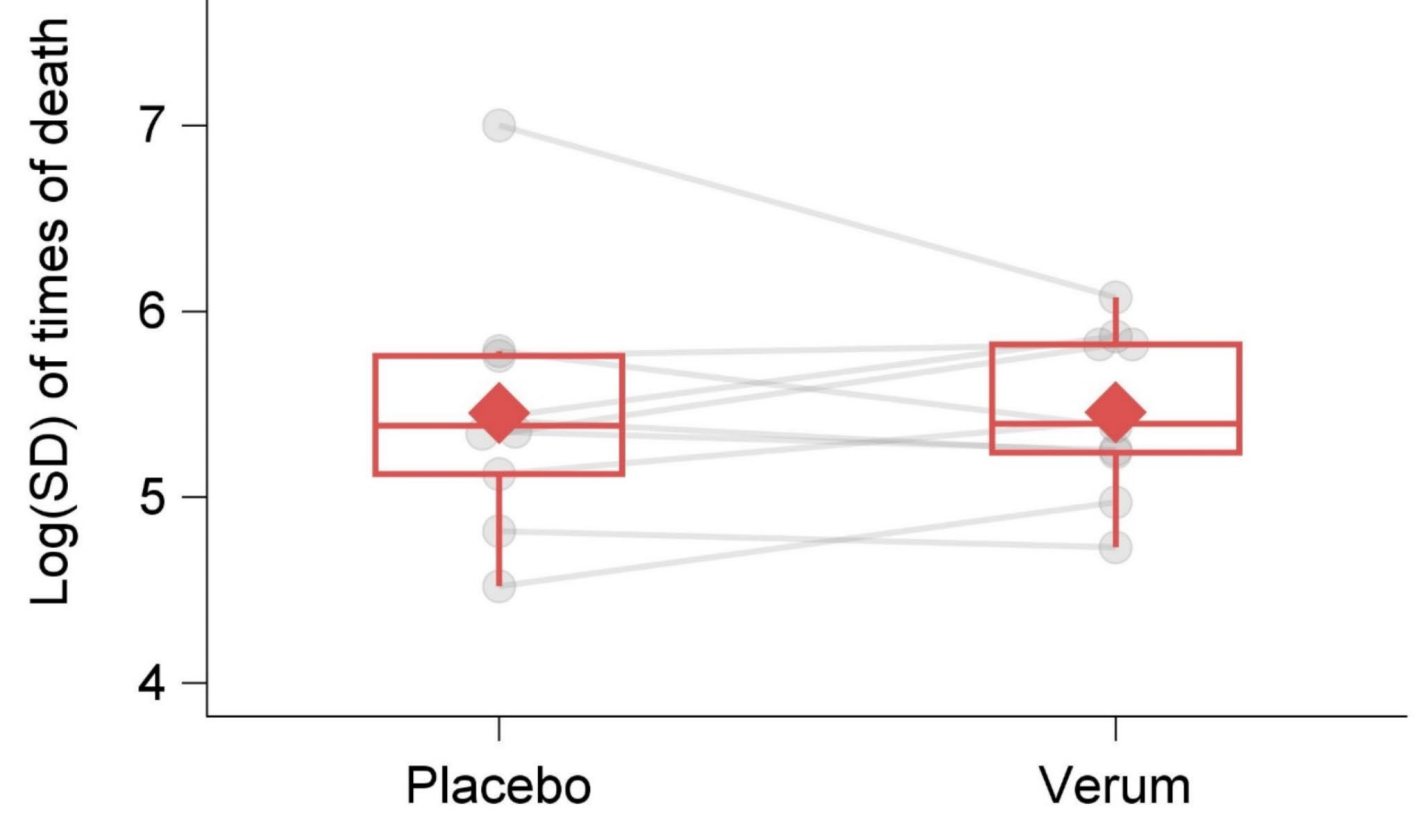
Observed values (gray points) and boxplots (red lines) for the log(SD) of time of death, separately for placebo and verum arms. The two observed values from the same trial are connected by a gray line. Bottom and top edges of a box display the first (Q1) and third (Q3) quartile, the line inside the box indicates the median value. The red diamond within a box shows the mean value. The whiskers that extend from a box indicate the range of values that are outside of the intra-quartile range. Note that these boxplots do not adjust for the mean time to death, the sample size or for the correlation within trials

The mean (median/minimum/maximum) log(SD) of time to death was 5.46 (5.39/4.73/6.07) across all verum arms, and 5.45 (5.38/4.52/7.00) across all placebo arms, see Fig. 1. After adjusting for the log(Mean) of time to death, the correlation of arms within trials and inverse-variance weighting, the difference of log(SD) between verum and placebo was −0.036 [95%-CI: −0.059; −0.013], indicating a slightly larger variability in the placebo arms. With respect to the existence of clinical predictors to explain treatment heterogeneity, no clinically relevant interactions between treatment and any of the clinical predictors was observed (see Additional file, Table S2 and Figs. S2, S3).

## Discussion

This meta-regression analysis, using the digitalized individual information on time to death from ten large CVOTs on DPP-4 inhibitors, GLP-1 receptor agonists, and SGLT-2 inhibitors, including 99,746 observations and 7563 deaths, showed no evidence for the potential of the precision treatment approach in type 2 diabetes with respect to all-cause mortality. On the contrary, if the observed difference between placebo and verum arms would be considered relevant, then the effect is more heterogeneous in the placebo arms. This finding of a low potential for precision treatment in type 2 diabetes confirms our previous findings regarding glycaemic control and body weight [6, 7].

One strength of our study is that we could use the individual information on all-cause mortality for all participants of 10 CVOTs to fit parametric distributions and derive the variability outcome of the log(SD) of time to death. Otherwise this analysis would have been impossible, as this log(SD) (or any other measure for the variability of time to death) was not reported in any of the original trial publications. Further, by using all-cause mortality as our clinical outcome here, we investigated the “hardest” possible clinical outcome with large clinical relevance for the treated individual. Moreover, all-cause mortality is the only clinical outcome that is not affected by competing risks.

In our previous work for the clinical outcome of HbA1c [6] we found a larger variability after verum treatment with GLP-1 receptor agonists in individuals with poor initial glycaemic control. This calls for a replication and/or validation of this finding with other clinical outcomes and/or with different study designs. In the analysis reported here the overall effect for GLP-1 receptor agonists with respect to the log(SD) was larger in the placebo arms (Additional file, Fig. S2, −0.050 [95%-CI: −0.080; −0.020]). When looking at the subgroup of GLP-1 receptor agonists trials, there is larger variability in the verum arms with higher HbA1c baseline values (Additional file, Fig. S4) that, however, reverses (−0.010 [95%-CI: --; --]) after full adjustment in the meta-regression model. Unfortunately, the number of observations, i.e. treatment arms, is too small (*N* = 6) here to give standard errors for the meta-regression estimate. Interestingly, no evidence for a differential effect of GLP-1 receptor agonists on all-cause mortality across different baseline HbA1c categories was also seen in two post-hoc analyses of the REWIND [24] and the EXSCEL trial [25].

A limitation of our study is that the applied approach does only give indirect evidence for the clinical benefit of the precision treatment approach. It is possible that there is real treatment heterogeneity although there are similar variabilities in placebo and verum arms. This would be the case when there would be a strong negative correlation between an individual’s potential outcome from the placebo and the verum treatment.

There is a parallel line of research with regard to the benefit of the precision medicine approach, which is subsumed under the heading of “heterogeneous treatment effects (HTE)”, see Kent [26] for a recent review, and Venkatasubramaniam et al. [27] for an application in type 2 diabetes treatment. These approaches use ideas of causal inference as well as prediction modelling to directly derive individualized treatment rules. However, individual participant data have to be available to derive them and these can be achieved at reasonable cost only from a small number of trials, potentially limiting external validity. This has to be contrasted with our approach that only needs summary data from the RCTs allowing to include information from a considerably larger number of trials. As such, our approach has to be considered not as an opponent, but as a supplement to the evaluation of precision medicine by the HTE approach.

We are not aware of other studies that use the described meta-regression model to assess the potential of the precision medicine idea in the treatment of type 2 diabetes. However, there have been similar investigations in other clinical disciplines, e.g., in psychiatry [28, 29], hepatology [30], or pain research [31]. In all of these studies, treatment effect heterogeneity was also observed to be rather low.

We have to further acknowledge that with respect to clinical predictors we had no access to the individual participant data but had to rely on summary measures for the respective trial populations. This comes with the risk of ecological bias, but has the advantage that the full individualized information from the CVOTs is not necessary.

It might be considered another limitation of this work that we only used information from the large CVOTs. This is due to the fact that for fitting the necessary Weibull distributions we must have access to individual participant data for time to death, and, without taking the extra effort of obtaining these data from the data owners, published Kaplan–Meier curves have to be available. In principle, it would be possible to derive Weibull parameters also from trials which give survival information for all treatment groups at least two time points and we showed in previous work that such information is available in principle [32]. However, these trials are rather small as compared to the CVOTs, and would thus be down-weighted in the meta-regression approach applied here, thus informing the overall difference of variabilities only to a small degree. In focusing on the large CVOTs we of course had to rely on the 8 reviews of Schnell et al. [8–15] to contain all large CVOTs, because we did not conduct a new systematic review.

We have finally to acknowledge that the large CVOTs include specific populations with high risk for cardiovascular outcomes and thus also all-cause mortality, so there might be treatment heterogeneity and potential for the precision treatment approach in populations with lower risk. In addition, the study duration of the CVOTs might be too short for treatment heterogeneity to emerge.

To summarize from a methodological point of view, we now analysed the potential of the precision medicine idea with regard to type 2 diabetes treatment for three different clinical outcomes (HbA1c [6], body weight [7], and all-cause mortality) with the described meta-regression method. The results were remarkably similar, suggesting a rather low potential. There is a possible avenue to use individual person data to assess the clinical benefit of the precision medicine approach. To identify treatment heterogeneity with individual data [33], however, specialized trial designs are needed, e.g. replicated crossover or N-of-1-trials, in which individuals are treated at least twice with at least one of the treatments under study. Up to now, such trials were rather rare and we are not aware of even a single trial that compares an anti-glycaemic drug to placebo in such a setting, for an example in hypertension treatment see Sundström et al. [34]. Thus, future work to assess the potential of precision treatment in diabetology should initiate trials using these designs.

In conclusion, the small differences in variabilities of time to death between verum and placebo arms of ten large CVOTs and the absence of predictors for treatment heterogeneity suggest a low potential for the precision treatment approach for reducing all-cause mortality in the treatment of type 2 diabetes, at least for DPP-4 inhibitors, GLP-1 receptor agonists, and SGLT-2 inhibitors. This is not necessarily a disadvantage for treated individuals because if treatments work similarly in all of them, then no one is treated inferior. For actual clinical practice it is thus safe to assume that the average treatment effect as observed in standard RCTs applies to the treated person, at least until falsification of our results with new data, from new designs, or for entirely new treatments. Actual choice of treatments in clinical practice can thus follow the treatment paths from recent guidelines (e.g [35]).

## Electronic supplementary material

Below is the link to the electronic supplementary material.

Supplementary Material 1

## Data Availability

The data for reproducing the primary meta-regression analysis are given in the accompanying online supplementary material (Additional file, Table S3). The digitalized individual participant data were already used in a previous project and are available on https://zenodo.org/record/6630421.
