# Supplementary material for: The potential of precision diabetology for type 2 diabetes treatment—evidence from a meta-regression for all-cause mortality from large cardiovascular outcome trials
Source: Acta Diabetol. 2024 Dec 12;62(7):1073–80. doi: 10.1007/s00592-024-02425-8 (PMC12283822; doi:10.1007/s00592-024-02425-8)

**Additional file**

**Table S1:** The Weibull distribution

The density of the Weibull distribution with a scale parameter *b* (*b* > 0) and a shape parameter *c* (*c* > 0) is

$f\left( x \right)=\frac{c}{b}\left( \frac{x}{b} \right)^{c-1}e^{{-\left( x/b \right)}^{c}}$,

and the survival function is

$S\left( x \right)=e^{{-\left( x/b \right)}^{c}}$.

The mean of a Weibull distribution is $b\Gamma\left( 1+\frac{1}{c} \right)$,

the variance $b^{2}\left[ \Gamma\left( 1+\frac{2}{c} \right)-\left( \Gamma\left( 1+\frac{1}{c} \right) \right)^{2} \right]$,

the SD $b\sqrt{\left[ \Gamma\left( 1+\frac{2}{c} \right)-\left( \Gamma\left( 1+\frac{1}{c} \right) \right)^{2} \right]}$,

the natural logarithm of the SD $ln\left( b\sqrt{\left[ \Gamma\left( 1+\frac{2}{c} \right)-\left( \Gamma\left( 1+\frac{1}{c} \right) \right)^{2} \right]} \right)$,

with $\Gamma$denoting the gamma function.

**Table S2:** Results from assessing the second prerequisite, existence of clinical predictors for the log(SD) of time to death. Each line reports on a separate meta-regression model for each clinical predictor. The models are identical to the models for the first prerequisite; however, they were extended by an additional interaction term of the respective predictor with treatment. Given are the slopes of regression lines for the respective predictor in the placebo and the verum arms, as well as their difference, which actually measures the interaction between treatment and predictor

| Clinical predictor | Number of missing arms for the predictor | Slope of adjusted regression line in: | | Slope difference (verum-placebo) (95%-CI) |
| --- | --- | --- | --- | --- |
|  |  | Verum arms (95%-CI) | Placebo arms (95%-CI) |  |
| Mean age at baseline (in years) | 0 | -0.005 (-0.058; 0.047) | -0.011 (-0.068; 0.045) | 0.006 (-0.011; 0.023) |
| Proportion of male participants at baseline (in %) | 0 | 0.005 (-0.008; 0.018) | 0.003 (-0.011; 0.016) | 0.003 (-0.003; 0.008) |
| Mean BMI at baseline (in kg/m^2^) | 0 | -0.078 (-0.137; -0.020) | -0.059 (-0.116; -0.001) | -0.020 (-0.055; 0.016) |
| Mean HbA1c at baseline (in %) | 0 | -0.005 (-0.201; 0.190) | 0.003 (-0.202; 0.207) | -0.008 (-0.064; 0.049) |
| Mean HbA1c at baseline (in mmol/mol) | 0 | -0.0005 (-0.0184; 0.0174) | 0.0002 (-0.0185; 0.0190) | -0.0007 (-0.0059; 0.0045) |
| Mean disease duration at baseline (in years) | 2 | 0.020 (-0.023; 0.063) | 0.021 (-0.025; 0.067) | -0.001 (-0.014; 0.013) |
| Mean eGFR at baseline (in mL/min/1.73 m^2^) | 0 | -0.010 (-0.016;-0.005) | -0.010 (-0.015; -0.004) | -0.001 (-0.004; 0.002) |
| Mean systolic blood pressure at baseline (in mmHG) | 4 | 0.047 (-0.002; 0.095) | 0.043 (-0.002; 0.087) | 0.004 (-0.011; 0.020) |
| Mean total cholesterol at baseline (in mg/dL) | 6 | -0.007 (-0.024; 0.012) | -0.005 (-0.024; 0.013) | -0.001 (-0.011; 0.010) |
| Mean triglycerides at baseline (in mg/dL) | 6 | 0.003 (-0.004; 0.011) | 0.003 (-0.005; 0.011) | 0.0005 (-0.0021; 0.0030) |
| Median follow-up time (in months) | 0 | -0.007 (-0.011; -0.002) | -0.005 (-0.010; -0.001) | -0.001 (-0.003; 0.001) |
| Year | 0 | -0.029 (-0.071; 0.014) | -0.021 (-0.062; 0.019) | -0.007 (-0.025; 0.011) |

**Table S3:** Full data set for the primary meta-regression model

| **Study** | **Treatment** | **Log(SD)** | **Log(Mean)** | **Weight** |
| --- | --- | --- | --- | --- |
| CANVAS | Placebo | 5.7585 | 5.9295 | 33.7118 |
| CARMELINA | Placebo | 4.8165 | 5.0320 | 48.2857 |
| CREDENCE | Placebo | 4.5224 | 4.9232 | 31.7180 |
| DECLARE-TIMI58 | Placebo | 5.7866 | 6.0101 | 40.1690 |
| EMPA-REG Outcome | Placebo | 5.3408 | 5.5381 | 19.8198 |
| EXAMINE | Placebo | 6.9988 | 6.7094 | 5.9318 |
| EXSCEL | Placebo | 5.4143 | 5.6496 | 64.0566 |
| LEADER | Placebo | 5.1223 | 5.4355 | 56.3290 |
| REWIND | Placebo | 5.3514 | 5.6355 | 79.4202 |
| TECOS | Placebo | 5.4317 | 5.6440 | 50.4252 |
| CANVAS | Verum | 5.8214 | 6.0110 | 47.9834 |
| CARMELINA | Verum | 4.7288 | 4.9805 | 50.7159 |
| CREDENCE | Verum | 4.9729 | 5.2684 | 19.3689 |
| DECLARE-TIMI58 | Verum | 5.3801 | 5.7322 | 47.8189 |
| EMPA-REG Outcome | Verum | 5.8197 | 5.9751 | 20.8246 |
| EXAMINE | Verum | 6.0730 | 6.0111 | 8.2658 |
| EXSCEL | Verum | 5.2399 | 5.5634 | 62.9815 |
| LEADER | Verum | 5.4091 | 5.6698 | 39.9100 |
| REWIND | Verum | 5.2536 | 5.6029 | 75.7100 |
| TECOS | Verum | 5.8635 | 5.9543 | 39.5412 |

**Figure S1:** Kaplan-Meier estimates of the extracted data with 95% confidence intervals of the Weibull fit for the respective maximum observation time of the trial


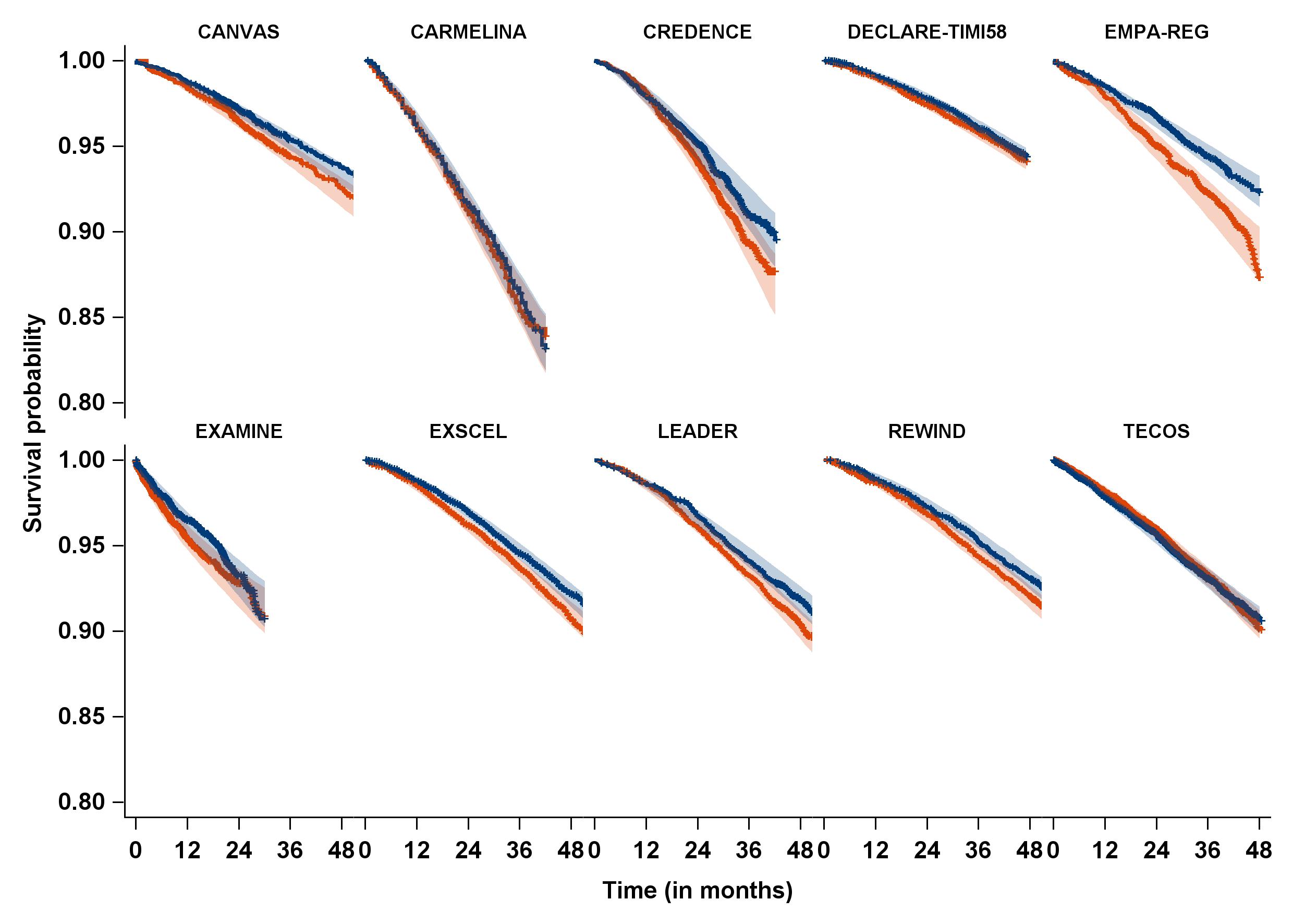


**Figure S2:** Scatterplots of the log(SD) of times to death against predictors (**a**) mean age at baseline, (**b**) proportion of male participants at baseline, (**c**) mean BMI at baseline, (**d**) mean disease duration at baseline, (**e**) mean HbA1c at baseline (in %), (**f**) mean HbA1c at baseline (in mmol/mol), (**g**) mean eGFR at baseline, (**h**) mean systolic BP at baseline, (**i**) mean total cholesterol at baseline, (**j**) mean triglycerides at baseline, (**k**) median follow-up time, and (**l**) year in the respective treatment arms. Weighted linear fits are given for both treatments, and the two linear regression lines being non-parallel would point to an interaction between the clinical predictor and treatment. The size (i.e. the surface area) of the bubbles is proportional to the sample size in the respective trial arm. Note that the linear fits account for the different weights of trial arms, but are not adjusted for mean times of death or the correlation within trials


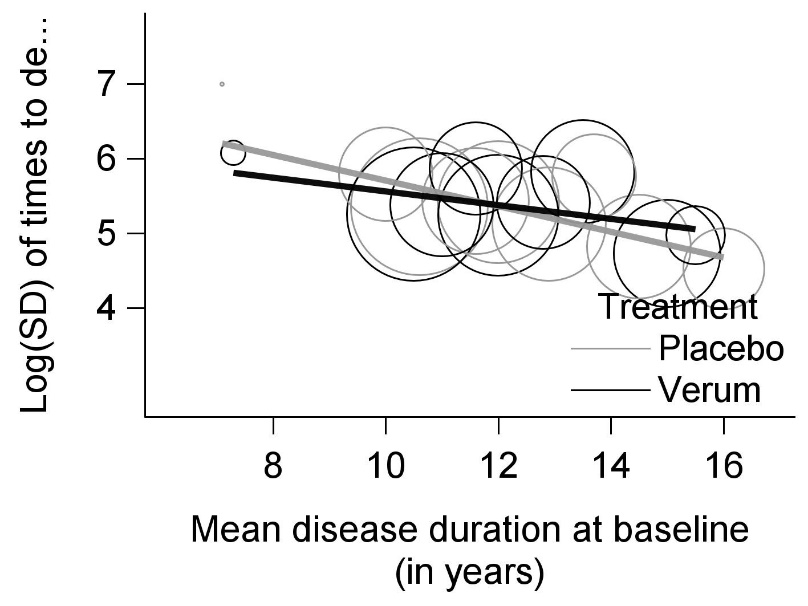

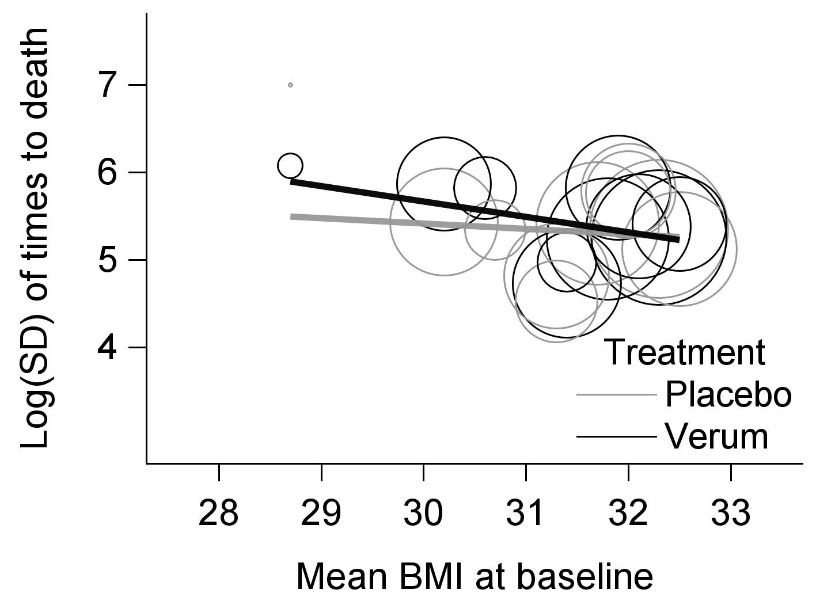

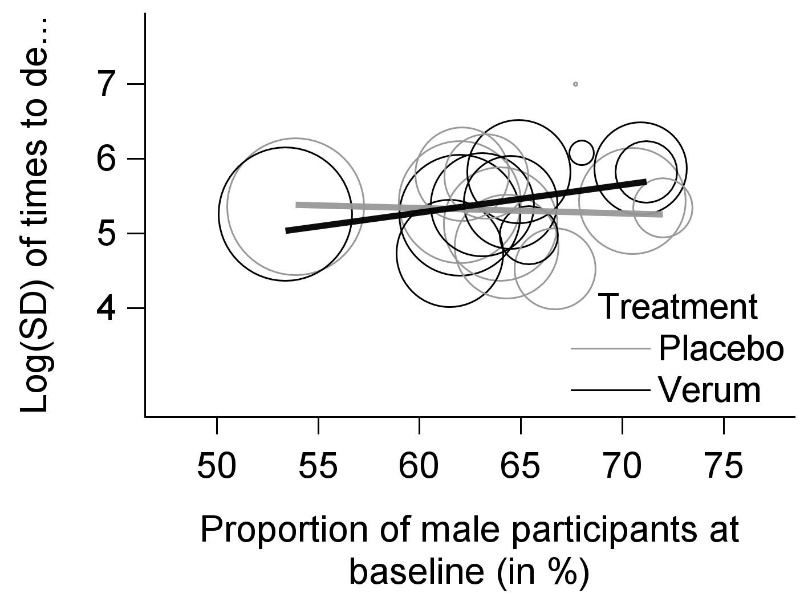

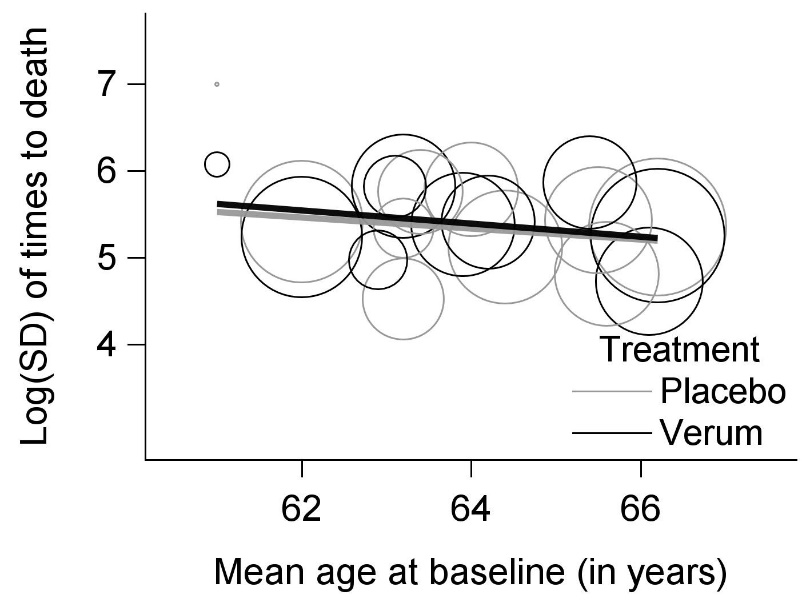


**d**

**c**

**b**

**a**


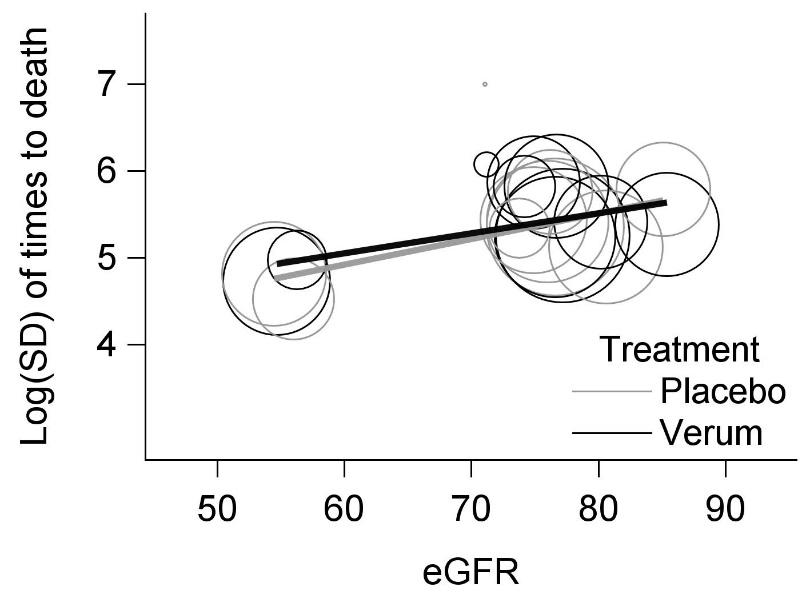

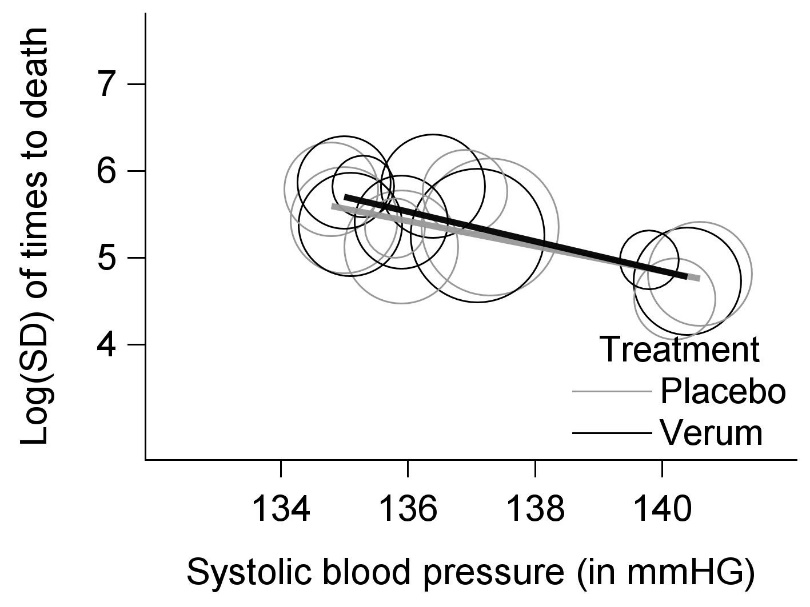

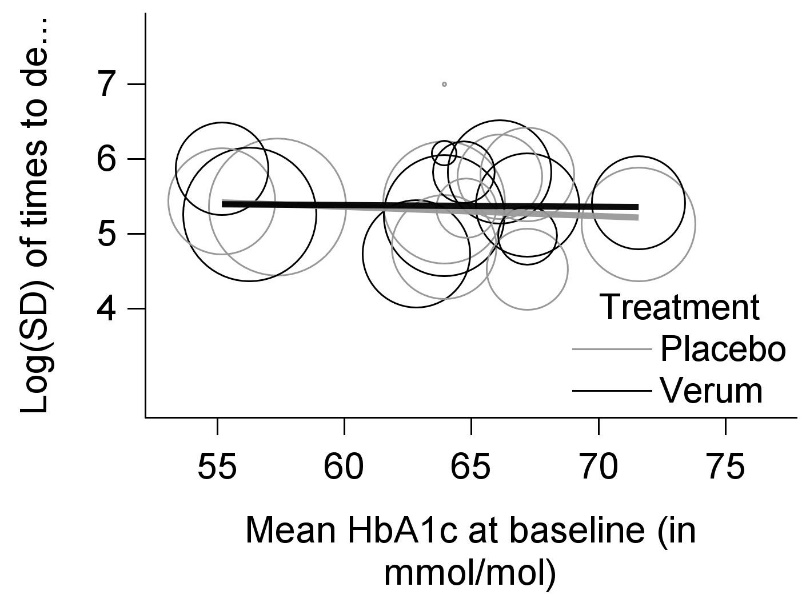

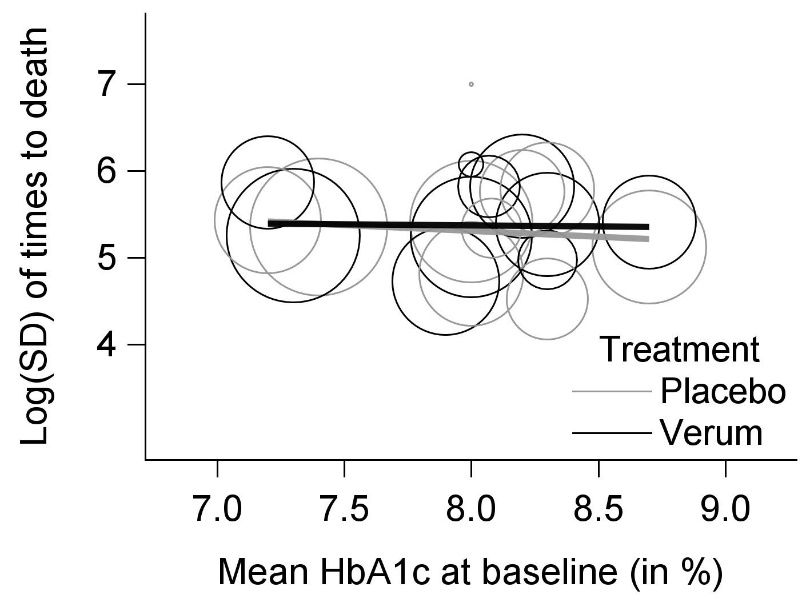


**e**

**f**

**g**

**h**

**
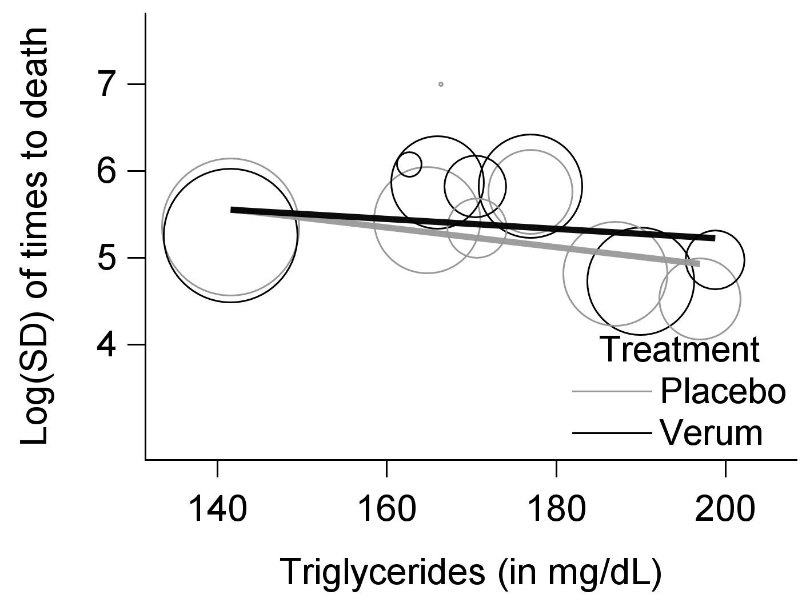

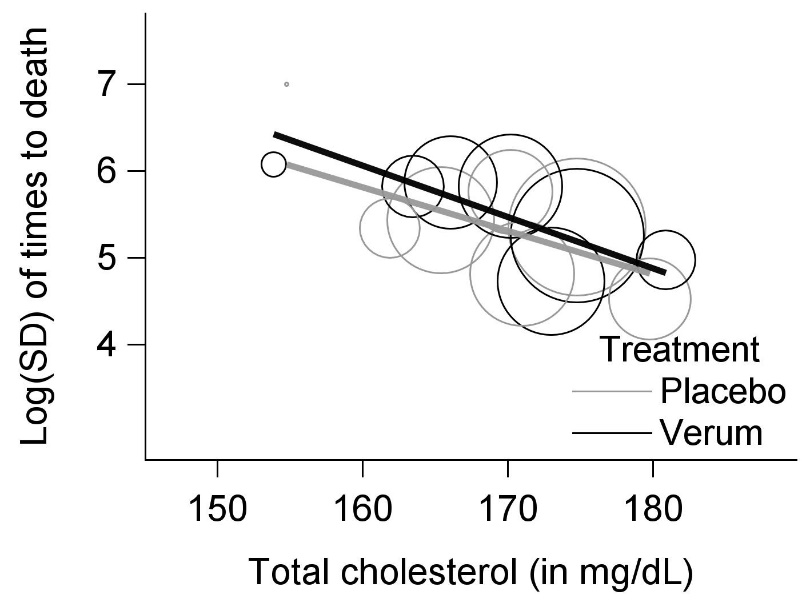

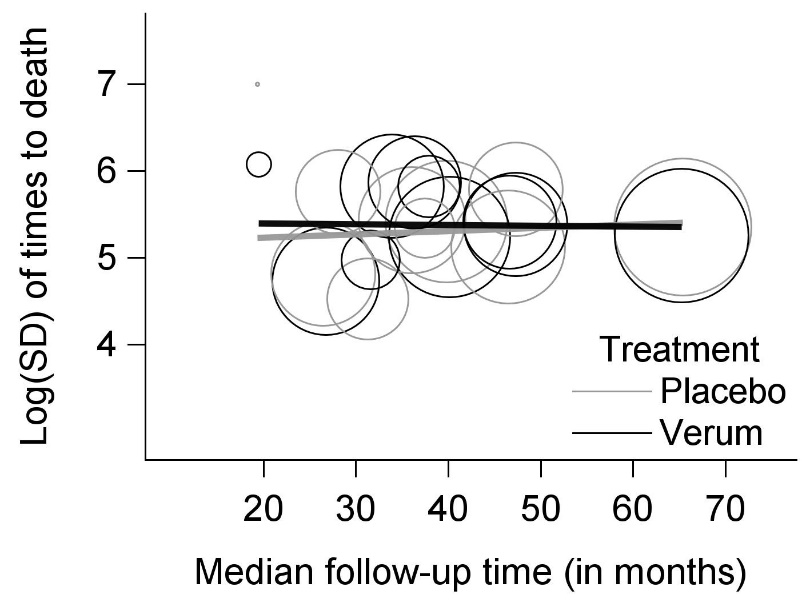

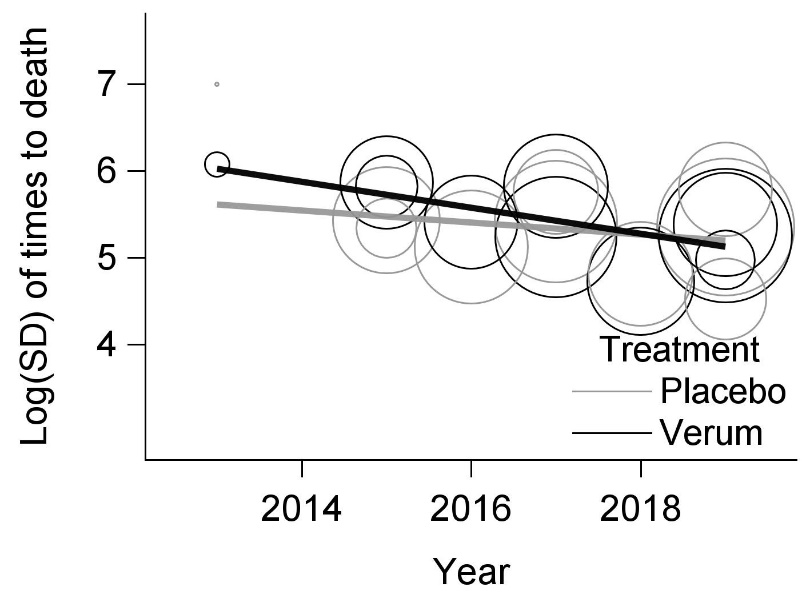
**

**i**

**j**

**k**

**l**

**Figure S3:** Differences in log(SD) of times to death against placebo for all treatments (drug classes)


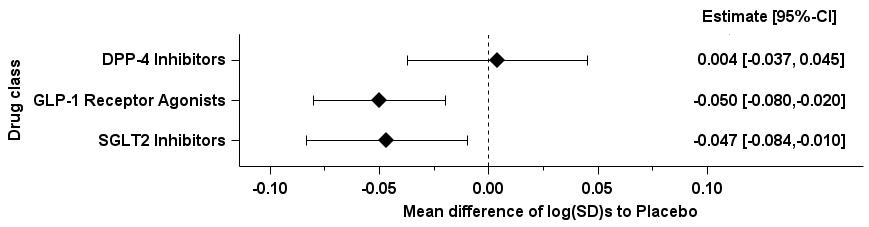


**Figure S4:** Scatterplot of the log(SD) of time to death against mean HbA1c at baseline (in %) for the subgroup of GLP1-RA trials. Weighted linear fits are given for both treatments, and the two linear regression lines being non-parallel would point to an interaction between the baseline HbA1c and treatment. The size (i.e. the surface area) of the bubbles is proportional to the sample size in the respective trial arm. Note that the linear fits account for the different weights of trial arms, but are not adjusted for mean times to death or the correlation within trials.


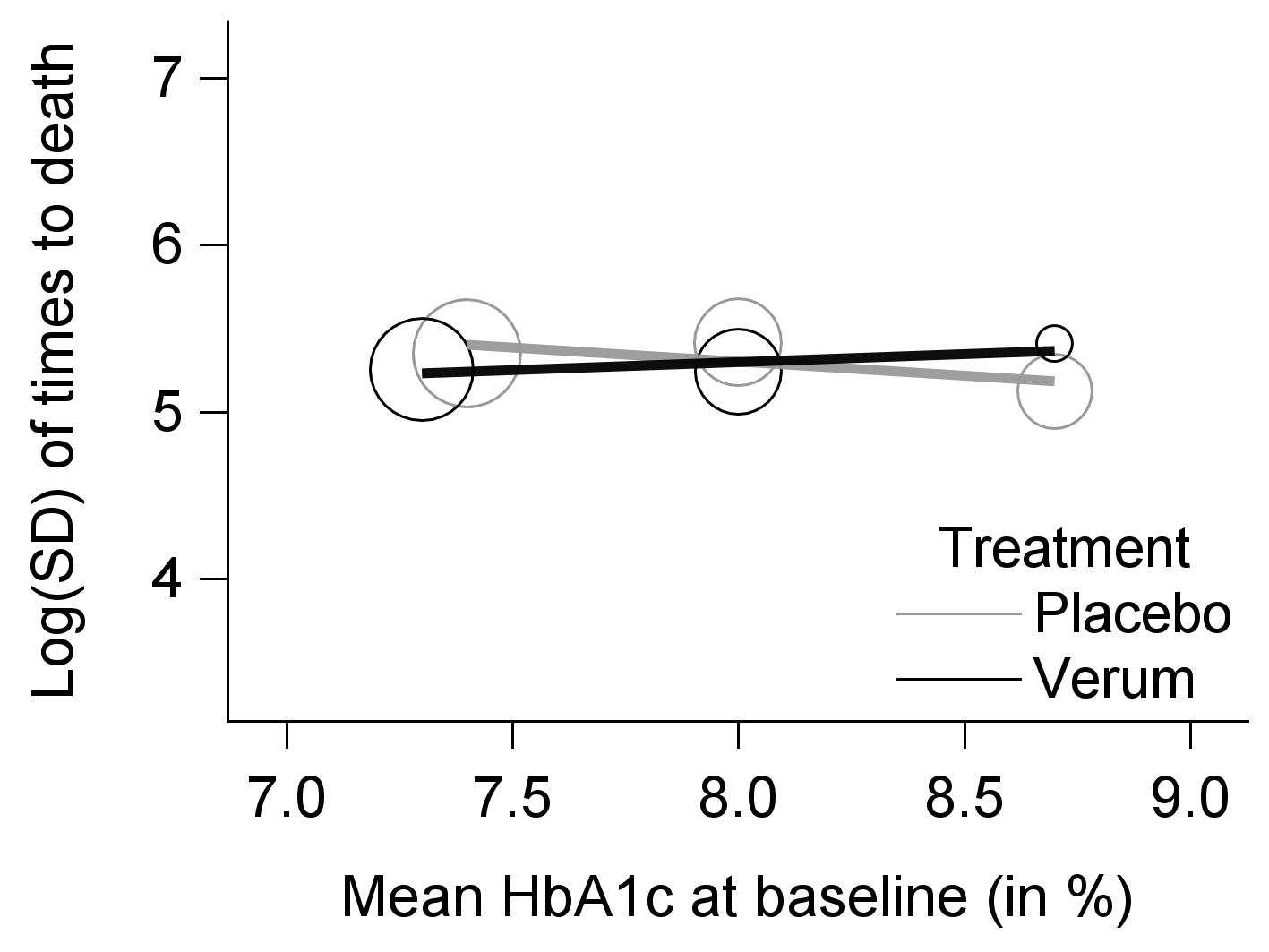

Supplement: Supplementary file 1 — Supplementary Material 1 [file 592_2024_2425_MOESM1_ESM.docx]
